# Feather corticosterone levels are related to age and future body condition, but not to subsequent fitness, in a declining migratory songbird

**DOI:** 10.1093/conphys/cow041

**Published:** 2016-10-04

**Authors:** Than J. Boves, Graham D. Fairhurst, Clark S. Rushing, David A. Buehler

**Affiliations:** 1 Department of Biological Sciences, Arkansas State University, Jonesboro, AR 72401, USA; 2 Department of Biology, University of Saskatchewan, Saskatoon, CanadaSK S7N 5E2; 3 Migratory Bird Center, Smithsonian Conservation Biology Institute, National Zoological Park, Washington, DC 20008, USA; 4 Department of Forestry, Wildlife and Fisheries, University of Tennessee, Knoxville, TN 37966, USA

**Keywords:** Carry-over effects, cerulean warbler, hormone biomarker, Neotropical–Nearctic migrant, non-breeding, *Setophaga cerulea*

## Abstract

In migratory species, breeding and non-breeding locations are geographically separate, yet the effects of conditions from one stage may carry over to affect a subsequent stage. Ideally, to understand the mechanisms and implications of ‘carry-over effects’, one would need to follow individuals throughout the year, quantify potential environmental causal factors and physiological mediators during multiple life-history stages, and measure downstream fitness. Owing to current limitations of tracking technology, this is impossible for small, long-distance migrants, so indirect methods to characterize carry-over effects are required. Corticosterone (CORT) is a suspected physiological mediator of carry-over effects, but when collected from blood it provides only a physiological snapshot at that point in time. When extracted from feathers, however, feather corticosterone (CORT_f_) provides a measure of responses to stressors from previous, and longer, time periods. We collected feathers grown during two life-history stages (post-breeding and subsequent wintering) from individuals of two age classes of a rapidly declining migratory songbird, the cerulean warbler (*Setophaga cerulea*), on their breeding grounds and quantified CORT_f_ concentrations. We then monitored reproduction and survival of individuals and analysed relationships among CORT_f_ and age, body condition and future fitness. Compared with older males, second-year males had higher CORT_f_ concentrations during both stages. When controlling for age and year, body condition at capture was positively related to CORT_f_ concentrations from winter (especially for older birds). However, we found no relationships between CORT_f_ and fitness (as defined by reproduction and survival). Thus, elevated CORT may represent a beneficial physiological response (e.g. hyperphagia prior to migration), particularly for certain life-history stages, and may mediate the condition in which individuals transition between stages. But for those birds that survive migration, subsequent fitness is likely determined by more recent events and local conditions (i.e. on breeding grounds), which have the potential to counteract conditions from the winter.

## Introduction

Animals face a variety of environmental challenges during different stages of their annual cycles. In migratory species, breeding and non-breeding locations are geographically separate, yet despite this, the effects of conditions experienced during one stage can carry over to affect performance during a subsequent stage (Norris and Marra, 2007; Harrison *et al*., 2011; Tonra *et al*., 2014). In order to gain a complete understanding of the mechanisms and implications of these ‘carry-over effects’, researchers need to follow individuals throughout the year and quantify potential environmental causal factors (e.g. food availability, pollutants) and physiological mediators (e.g. body condition, stress physiology) during multiple life-history stages, as well as measure downstream fitness effects themselves (i.e. future reproduction and survival). Carry-over effects have been directly quantified for some larger resident or short-distance migrants (e.g. Salton *et al*., 2015), but this is virtually impossible for many species, particularly small migratory birds that make rapid, long-distance migrations and cannot be monitored on a daily basis throughout their entire life cycle (e.g. Stutchbury *et al*., 2009; DeLuca *et al*., 2015). As a result of this, few studies have been able to relate physiological correlates of environmental conditions faced by small migratory birds from multiple life-history stages to subsequent components of fitness at the individual level. This is an important shortcoming in our understanding of how populations are limited, because many species of small migratory songbirds increasingly face human-caused threats throughout the annual cycle (Webster and Marra, 2005), yet most research has focused only on the breeding period (Marra *et al*., 2015).

The main proposed mechanism of carry-over effects is non-lethal variation in physiological ‘state’ attributable to environmental factors (Harrison *et al*., 2011), such as diet (Sorensen *et al*., 2009; Kouwenberg *et al*., 2013), especially in low-quality habitats (Marra *et al*., 1998). As it is often difficult to measure physiological state directly, short-term measures, such as body condition indices and blood concentrations of the hormone corticosterone (CORT), have been used as indirect measures. Corticosterone is especially relevant to carry-over effects because it is integral to energy management and is released in quantity upon exposure to challenging or threatening environmental conditions (i.e. stressors), such as low food availability, predators, anthropogenic disturbances or poor weather (i.e. the purported drivers of carry-over effects; Wingfield *et al*., 1983; Marra and Holberton, 1998; Wingfield and Kitaysky, 2002; Kitaysky *et al*., 2010; Harrison *et al*., 2011; and see Fairhurst *et al*., 2015a). By orchestrating changes in physiology and behaviour aimed at maximizing survival, CORT provides a hypothesized functional link between environmental variation and fitness.

Corticosterone-mediated responses to environmental conditions during the non-breeding stage may underlie carry-over effects to the breeding stage in migratory birds. Limited previous work supports this hypothesis. For example, American redstarts (*Setophaga ruticilla*) that wintered in higher-quality habitats in Jamaica had, on average, lower levels of baseline CORT (Marra and Holberton, 1998), departed from wintering grounds and arrived on breeding grounds earlier and in better condition (Marra *et al*., 1998), fledged more young (Norris *et al*., 2004) and returned to wintering grounds at greater rates (Studds and Marra, 2005). Likewise, in Arctic-breeding common eiders (*Somateria mollissima*), birds with higher CORT during the non-breeding stages arrived later on the breeding grounds and were in poorer body condition, which led to poorer reproductive performance (Harms *et al*., 2015). In contrast, however, Atlantic Puffins (*Fratercula arctica*) with elevated CORT during the non-breeding stage subsequently laid larger eggs (Kouwenberg *et al*., 2013), which is typically related positively to fitness (Krist, 2011). These studies, with their contrasting results, suggest that: (i) CORT during the non-breeding stage can mediate carry-over effects into the breeding stage; but (ii) the directionality of the relationship between CORT and future performance traits may be context dependent. Moreover, physiological responses to stressors may co-vary with other factors that play important roles in future survival or reproduction. For example, younger individuals may struggle to locate food because they may be naïve to their non-breeding environment (Wunderle, 1991; Daunt *et al*., 2007) and thus may elevate CORT concentrations to increase survival (Lendvai *et al*., 2015), but they may fail to reproduce successfully because of lack of experience, regardless of their level of stress (Martin, 1995; Hawkins *et al*., 2012). However, to our knowledge no previous study has assessed individual-level relationships between CORT from non-breeding and subsequent reproduction in multiple age groups, so our understanding of the role of age in influencing carry-over effects remains limited.

In this study, we hypothesized that CORT during non-breeding mediates carry-over effects to the breeding season and that this relationship is age dependent. We tested this in a long-distance avian migrant, the cerulean warbler (*Setophaga cerulea*), a songbird with severely declining populations in North America (Buehler *et al*., 2013, Sauer *et al*., 2014). By analysing CORT from tail and rump feathers (hereafter, CORT_f_) collected from males on the breeding grounds, we retrospectively quantified physiology from two life stages: the prior non-breeding stage (rump) and the post-breeding/post-fledgling stage that preceded it (tail). This allowed us to determine whether there was individual consistency in CORT_f_ during two stages of the annual cycle leading up to reproduction. We then related CORT_f_ from these previous stages to body condition, reproduction and apparent survival of the same individuals collected subsequently on the breeding grounds, while considering two potentially confounding factors of both CORT and reproduction (age and year).

We tested three predictions related to our hypothesis. First, compared with older (after-second-year; ASY) males, we expected younger (second-year; SY) males to have greater CORT_f_ in both life-history stages (post-breeding/post-fledgling and non-breeding) and, subsequently, poorer body condition on breeding grounds, reflecting a lack of dominance and experience and, potentially, the use of lower-quality habitat. Second, we expected a relationship between CORT_f_ during non-breeding and subsequent body condition during breeding in both age classes, but recognize that the direction of this relationship may be context dependent (e.g. age dependent). Experimentally testing the mechanism(s) underlying such an association would be logistically impossible, so we explored this relationship correlatively, predicting that the relationship would be negative if poor condition during non-breeding and/or use of poorer-quality non-breeding habitats led to physiological and/or behavioural costs (e.g. poor condition prior to spring migration and/or delayed spring migration) associated with elevated CORT. In contrast, we would expect a positive relationship if good condition and/or use of good-quality non-breeding habitats allow birds to benefit from the behavioural effects or physiological results of elevated CORT during that time (e.g. increased hyperphagia and improved pre-migratory condition and/or earlier departure). Third, we expected CORT_f_ to correlate with one or more fitness parameters, but this could also be context dependent. If higher concentrations of CORT reflect poor body condition, then males with higher CORT_f_ may be paired less frequently, initiate nesting later, be less likely to breed successfully, produce fewer young and/or be less likely to return. Alternatively, if higher CORT reflects individuals in better condition, then males with higher CORT_f_ would be more likely to pair, initiate nesting earlier, breed successfully, produce more young and/or be more likely to return the following year.

## Materials and methods

### Study species

Cerulean warblers are small Nearctic–Neotropical migratory birds that nest in the canopy of mature deciduous forests of eastern USA (Buehler *et al*., 2013) and are typically socially monogamous (but see Boves and Buehler, 2012), obligately single brooded, and will attempt to re-nest up to two times after failure (Buehler *et al*., 2013). On their wintering grounds in the northern Andes of South America (from north Colombia and Venezuela to northwest Bolivia >500 m above sea level), they use a variety of habitats, including primary and secondary forest (Buehler *et al*., 2013) as well as agroforestry plantations (Bakermans *et al*., 2009). Over the past 50 years, the global population of cerulean warblers has declined >3%/year (Sauer *et al*., 2014), and they are considered vulnerable, or a species of conservation concern, by many agencies and organizations (Buehler *et al*., 2013). The cause of this precipitous decline is not entirely clear, but a number of broad factors have been proposed, including a decrease in habitat quality on the breeding grounds (Boves *et al*., 2013) and a decrease in habitat quantity and quality on the wintering grounds (Buehler *et al*., 2013).

### Moult location in cerulean warblers

Although only minimal direct evidence of moult location in cerulean warblers exists (for either tail or rump), and we cannot be certain that every feather that we analysed was grown in the location we assume, the most likely pattern of moult (for individuals from both age classes) is that: (i) rectrices are moulted on the post-breeding grounds (or, less likely, during migration), and (i) rump feathers are moulted on their Andean wintering grounds. We use evidence from stable isotopes, plumage colouration, feather wear and migration timing to make these conclusions and describe each below.

#### After-second-year rectrices

Analysis of hydrogen stable isotopes suggests that tail feathers for ASY birds are grown on the breeding grounds or, for a small proportion of these older birds, early during migration (before leaving the USA; C.S.R., unpubublished data). In our estimation, moult-migration is more unlikely because of the high energetic cost of both of these activities and because cerulean warblers appear to be both early (often leaving the USA in early August) and rapid migrants (T.J.B., unpubublished geolocator data, personal observation).

#### Second-year rectrices

Juvenile cerulean warblers grow tail feathers that resemble SY males by 30 days post-fledging. Upon returning to the breeding grounds, tail feather wear is substantial, suggesting that these feathers have not been moulted more recently, i.e. on the wintering grounds (T.J.B., personal observation; D. W. Raybuck, personal communication, unpubublished data).

#### Second-year rump

Shortly after fledging, and into their first migration, juvenile and hatch-year (HY) male cerulean warblers possess body plumage that is different in colour from definitive adult male plumage (greyer and greener, similar to female; Pyle, 1997). The rump feathers we analysed, from both SY and ASY birds, were much bluer than this juvenile male plumage (Boves *et al*., 2014), suggesting a wintering grounds origin of the rump feathers. Hydrogen stable isotopes also support the suggestion that these feathers are grown on wintering grounds or during migration (C.S.R., unpubublished stable isotope analysis data). Moult-migration is more unlikely because of the same limitations described above.

#### After-second-year rump

Hydrogen stable isotope analysis suggests that these feathers are grown either on wintering grounds or during migration (C.S.R., unpublished data). Moult-migration is more unlikely because of the same limitations described above. No plumage or feather wear characteristics are useful in identifying the moult location for these feathers from ASY birds, but body moult has been documented during the winter in this age class (F. Newell, personal communication) and, to our knowledge, has not been observed during post-breeding or migration.

### Study area

We studied cerulean warblers from 2008 to 2010 on ~160 ha in the North Cumberland Wildlife Management Area, Campbell County, TN, USA (36°12′N, 84°16′W and 36°21′N, 84°18′W). Cerulean warblers are found at some of their greatest breeding densities in this region (Buehler *et al*., 2006); mean density of cerulean warblers ranged from 0.67 to 0.74 territories/ha/year (T.J.B., unpubublished data). The forest type was mostly mixed mesophytic, and tree composition was predominantly oaks (*Quercus* spp.), maples (*Acer* spp.), hickories (*Carya* spp.) and tulip poplar (*Liriodendron tulipifera*).

### Field methods

#### Capture, feather collection and morphometrics

We captured male cerulean warblers during the breeding season, from early May to mid-June. To capture individuals, we broadcast territorial songs and call notes and displayed a male conspecific decoy strategically set near a mist net. After capture, we aged birds as second-year (SY; i.e. in their first breeding season) or after-second-year (ASY; i.e. in or after their second breeding season) on the basis of moult limits and feather wear. Like many warblers species, SY male cerulean warblers retain some brownish juvenile alular feathers and primary coverts, and possess worn and pointed rectrices (Pyle, 1997). Once captured, we banded each bird with a unique combination of a federally issued aluminum leg band and one to three plastic coloured leg bands to enable later identification of individuals in the field without recapture; we also collected five rump feathers and one tail feather (right innermost rectrix). Finally, we measured right wing chord to the nearest 0.5 mm (using a straight wing rule) and mass to the nearest 0.1 g (using a digital scale).

#### Pairing, nest searching and monitoring

For all birds, we searched each territory for a minimum of 4 h/week during the entire breeding season (from May to early July) to determine pairing status and to search for nests. In addition, we conducted intensive spot-mapping sessions on eight mornings during the peak of the breeding season. We considered a male to be paired in the following circumstances: (i) if a nest was constructed within the territory of the male; (ii) if no nest was located, but a female was observed repeatedly within the male's territory; or (iii) if we observed male behaviour that would strongly suggest pairing (e.g. male carrying nesting material or food items to young). Despite identifying these criteria for pairing *a priori*, we successfully located nests for every paired individual in our sample and thus did not ever use criteria (ii) or (iii) to determine pairing. We used behavioural cues to find nests (e.g. nest material collection, building, foraging/provisioning), and we found the majority of nests (>75%) during the building stage. After a nest was located, we monitored it every 1–3 days to determine its fate. If nestlings survived to 7 days of age, we monitored nests daily for ~45 min with a spotting scope equipped with a ×60 eyepiece. We then counted nestlings daily (this was most effective when parents came to feed) until fledging, and on the day of fledging, we searched for fledglings in the area surrounding the nest to confirm success or failure. If a nest was successful, we considered the number of nestlings on the last day in the nest (typically day 9 or 10) to be the number of young fledged. Between days 7 and 10 of the nestling stage, we also video-recorded nests for 2 h (between 06.30 and 10.00 h) to confirm nestling counts and estimated male provisioning rates per nestling/h; we did not record nests on mornings with high winds or precipitation. When a nest failed, we returned to the territory and searched for evidence of re-nesting in the same manner used to find initial nests. Re-nesting attempts were often easier to locate because of the increased rate of building and the re-use of previous nesting material. We continued this process for all territories until the end of the breeding season (mid-July).

#### Annual survival

To assess future (apparent) survival, we returned to sites in subsequent years to resight colour-marked males across all the 160 ha study area and in adjacent areas ≤200 m of study area boundaries. In >8 years of studying this species in this area, nearly all (98%) known returning birds have settled within 100 m of previous season territorial boundaries (T.J.B. and D.A.B., unpubublished data), so we believe the 200 m buffer was sufficient to resight most individuals that returned to the general vicinity.

#### Estimates of body condition

We estimated body condition at capture from simple body mass measurements (using a digital scale). Although body mass can vary over time, we have found that during the breeding season there is little change in mass within individuals (T.J.B., unpublished data), but we did account for date of capture by including it as a random effect in our models. Body mass is often as or more reliable than unverified indices (Schamber *et al*., 2009), but we also evaluated an index of body condition by regressing mass on wing length and used the resulting residuals (Schulte-Hostedde *et al*., 2005).

### Feather corticosterone analysis

Extraction of CORT from feathers followed Bortolotti *et al*. (2008), which has been replicated with a variety of passerine species (Fairhurst *et al*., 2014, 2015b; Grunst *et al*., 2015). Given that we collected multiple rump feathers from each bird, we analysed two rump feathers per individual to ensure that hormone concentrations were detectable. Only a single rectrix was analysed for each bird because we collected only one per individual. We measured the length of each feather and, for rectrices, we removed the calamus before extraction; for the much smaller rump feathers, the calamus was minute, and proper removal (i.e. distal to the superior umbilicus) was not possible, so we included it in the samples. We cut each sample into pieces <5 mm^2^ with scissors, added 10 ml of methanol (HPLC grade; Fisher Scientific, Fairlawn, NJ, USA), and placed the samples in a sonicating water bath at room temperature for 30 min, followed by incubation at 50°C overnight in a water bath. We separated the methanol extract from feather material by vacuum filtration, using a plug of synthetic polyester fibre fitted in a filtration funnel. We then placed all methanol extracts in a 50°C water bath and evaporated them in a fume hood. We reconstituted extract residues in a small volume of phosphate-buffered saline (0.05 M, pH 7.6), transferred them to Eppendorf tubes, and froze them at −20°C. We assessed the efficiency of the methanol extraction by spiking three feather samples with a small amount (~5000 counts/min) of ^3^H-corticosterone in the extraction. We recovered 97.9% of the radioactivity in the reconstituted samples. All samples were extracted in a single batch.

The CORT_f_ concentrations were determined by radioimmunoassay following Wayland *et al*. (2002) and used a commercially available antibody (Sigma-Aldrich, St Louis, MO, USA; catalogue no. C8784). Measurements were performed in duplicate on reconstituted methanol extracts, and serial dilutions of samples were parallel to the CORT standard curve. Samples were measured in two assays, and the variability of each assay was assessed using six replicates of an internal standard. The average intra-assay coefficient of variation was 9.0% (SD = 2.1), and inter-assay coefficient of variation was 5.8%. The assays had an average detectability limit (%*B*/*B*_0_) of 21.1 pg CORT (SD = 4.8), but all samples were well above detectability limits. Data values are expressed as picograms of CORT per millimetre of feather, which gives a valid estimate of CORT per unit time of feather growth (Bortolotti *et al*., 2008; Bortolotti, 2010). Resulting CORT_f_ concentrations, for both feather types, were not correlated with sample feather mass within either age class (rump ASY, *F* = 0.36, d.f. = 1,37, *P* = 0.55; rump SY, *F* = 0.76, d.f. = 1,19, *P* = 0.39; rectrix ASY, *F* = 0.59, d.f. = 1,40, *P* = 0.45; and rectrix SY: *F* = 0.79, d.f. = 1,17, *P* = 0.39), suggesting that any differences in CORT_f_ concentrations between age classes were not attributable to the potential influence of feather mass. The CORT assays were performed at the University of Saskatchewan, Canada.

### Statistical analyses

Recent data suggest that CORT_f_ values are similar between different feather types (Lendvai *et al*., 2013), feathers grown on opposite sides of the bird (e.g. left and right wing), and adjacent feathers of the same type (see Romero and Fairhurst, 2016 for a review). However, given that the rump and rectrix feathers in our study were grown at different places and in different seasons (see ‘*Moult location in cerulean warblers*’ above), we did not expect these different feather types to reflect similar concentrations of plasma CORT. Thus, we avoided making direct comparisons between rump and rectrix CORT_f_ values in our study, and conducted separate statistical analyses for each feather type.

#### Relationship between feather corticosterone, age and year

We assessed the relationship of age and year with CORT_f_ by constructing general linear models, with CORT_f_ concentration as the response variable, and age, year and age × year as predictor variables. We examined distributional plots of univariate variables as well as residuals and tested for violations of assumptions of normality (Shapiro–Wilk *W* test) and constant variance (Levene's test). We found no violations for rump feathers, but for rectrices the data were positively skewed. We corrected the skewness by a reciprocal transformation of rectrix CORT_f_, which was applied to all subsequent models using this variable. For both feather types, if the interaction term was not significant (*P* > 0.05), we removed this term and re-ran the simplified models.

To determine whether there was individual consistency in CORT_f_ values between seasons, we assessed the relationship between rump CORT_f_ and rectrix CORT_f_ using a general linear mixed model with bird identity included as a random effect. This model used rump CORT_f_ as the response variable (because it was grown after the rectrix) and included an effect of age and a rectrix CORT_f_ × age interaction term as predictor variables. We were not interested in annual differences in this relationship and instead wanted to know whether there was a general relationship across all years, so we included year as a random term in this model. We included individual bird identity as a random term to account for the fact that we measured rump CORT_f_ and rectrix CORT_f_ in the same individuals.

#### Relationship between feather corticosterone and body condition metrics

We evaluated potential carry-over effects of CORT on two response variables related to body condition, namely body mass and a body condition index derived from body mass–wing length residuals. Each response variable was modelled separately with a general linear mixed model and included the predictors of rump CORT_f_, age, and a rump CORT_f_ × age interaction term. Year was included as a random effect in the model because we were not interested in annual differences but instead were concerned with whether the relationship between body condition and rump CORT_f_ was a general effect across years. Date of capture was also included as a random effect in the model because birds were captured through the breeding season, and we wanted to generalize our results across all potential capture dates. After first running saturated models, we removed non-significant interaction terms (*P* > 0.05) and re-ran the models in more simplified forms.

#### Relationship between feather corticosterone and fitness parameters

We evaluated the relationship between non-breeding CORT and future reproduction and survival of individuals by testing the relationship between rump CORT_f_ and several response variables related to fitness: first egg date (running date, May 1 = 1), number of fledglings produced (over entire season), pairing success (yes/no; Y/N), nesting success (Y/N), provisioning rate (no. of visits/h/nestling) and apparent annual survival (Y/N). For each response variable, we constructed generalized linear mixed models, which included the predictors of rump CORT_f_, age, and a CORT_f_ × age interaction. Year was included as a random effect in all models except for annual survival, where the model would not converge with mixed effects, so we included year as a fixed effect. We examined residuals and tested for violations of assumptions appropriate to the type of model used (normal or binomial distribution). To meet assumptions of normality and constant variance of residuals, we logarithmically transformed first egg date and the number of fledglings produced (after adding a constant of 0.5 to each value to allow logarithmic transformation of zeros). After first running saturated models, we removed non-significant interaction terms (*P* > 0.05) and re-ran the models in more simplified forms. Finally, to ensure that habitat conditions did not confound results, we also included average territory basal area (in square metres per hectare) to control for the potential effects of breeding habitat variability on reproductive measures. If inclusion of basal area had no impact on the statistical relationship between CORT_f_ and fitness measures (i.e. did not change the statistical significance in either direction), we removed this variable from the models. All statistics presented were derived from final models. Sample sizes vary by response variable for two reasons: (i) we censored some individuals if their inclusion in the analysis was illogical (e.g. we did not include unpaired individuals or those whose nests failed earlier in provisioning analysis); and (ii) we were uncertain about some information for individuals (e.g. unsure whether an individual reproduced successfully). All statistical analyses were conducted using SAS or JMP (SAS Institute, Cary, NC, USA). Untransformed means are presented ± 1 SEM.

## Results

### Relationship between feather corticosterone, age and year

**Figure 1:**
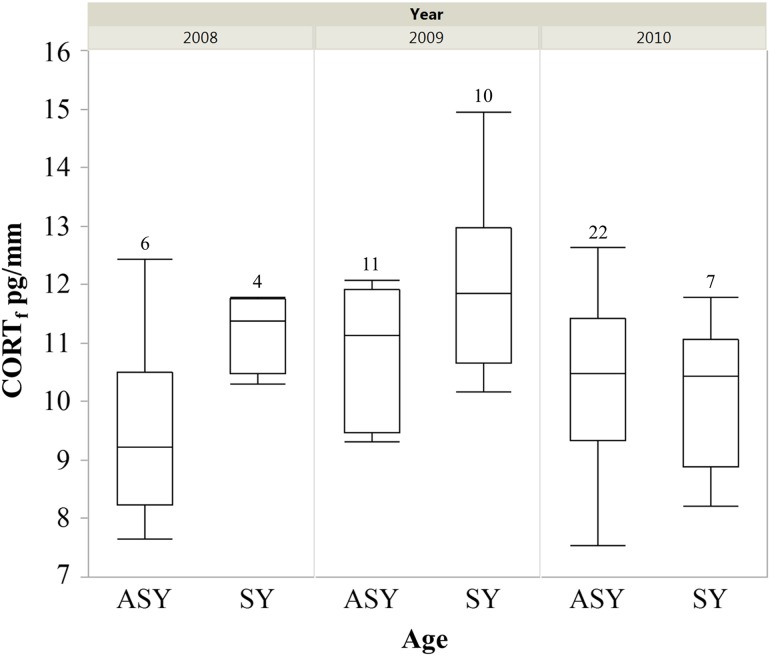
Box plots displaying concentrations of corticosterone in rump feathers (CORT_f_) of male cerulean warblers from two age classes collected from 2008–10 in the Cumberland Mountains, TN, USA. The CORT_f_ concentration differed between age classes when controlling for year (χ^2^ = 5.83, d.f. = 1,54, *P* = 0.02). ASY, after-second-year; SY, second-year.

**Figure 2:**
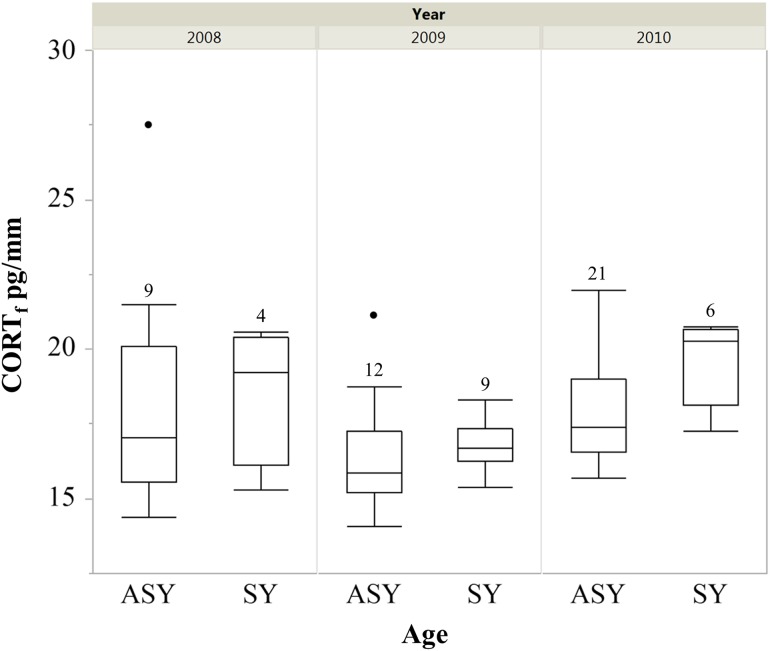
Box plots displaying concentrations of corticosterone from rectrix feathers (CORT_f_) of male cerulean warblers from two age classes collected from 2008–10 in the Cumberland Mountains, TN, USA. The CORT_f_ concentration differed between age classes when controlling for year (χ^2^ = 3.81, d.f. = 1,57, *P* = 0.05). ASY, after-second-year; SY, second-year. Sample size is indicated above the bars.

For rump feathers (grown on wintering grounds), young (SY) male cerulean warblers had greater CORT_f_ values than older (ASY) males when controlling for year (SY, *n* = 21, $\bar{x}$ = 11.26 ± 0.35 pg/mm; and ASY, *n* = 39, $\bar{x}$ = 10.34 ± 0.21 pg/mm; χ^2^ = 5.83, d.f. = 1,54; *P* = 0.02; Fig. 1). The CORT_f_ concentrations also varied annually when controlling for age (χ^2^ = 9.96, d.f. = 2,54, *P* = 0.01; Fig. 1) and the interaction of age × year was also significant (χ^2^ = 5.94, d.f. = 2,54, *P* = 0.05); however, this interaction appears minimal (see Fig. 1). For rectrix feathers (grown on post-breeding grounds), SY males had greater CORT_f_ concentrations (reciprocally transformed) than ASY males (SY, *n* = 19, $\bar{x}$= 18.04 ± 0.43 pg/mm; and ASY, *n* = 42, $\bar{x}$=17.46 ± 0.37 pg/mm; χ^2^ = 3.81, d.f. = 1,57, *P* = 0.05; Fig 2). CORT_f_ also differed by year when controlling for age (χ^2^ = 10.98, d.f. = 2,57, *P* = 0.004; Fig 2), but the interaction was not significant (χ^2^ = 1.28, d.f. = 2,55, *P* = 0.52) and was removed from the final model.

We failed to find individual consistency between CORT_f_ in rump CORT_f_ and rectrix CORT_f_ (*F* = 2.53, d.f. = 1,15.8, *P* = 0.13). The rectrix CORT_f_ × age interaction was not significant (*F*_1,49_ = 2.38, *P* = 0.13) and was removed from the final model.

### Relationship between non-breeding feather corticosterone and body condition

**Figure 3:**
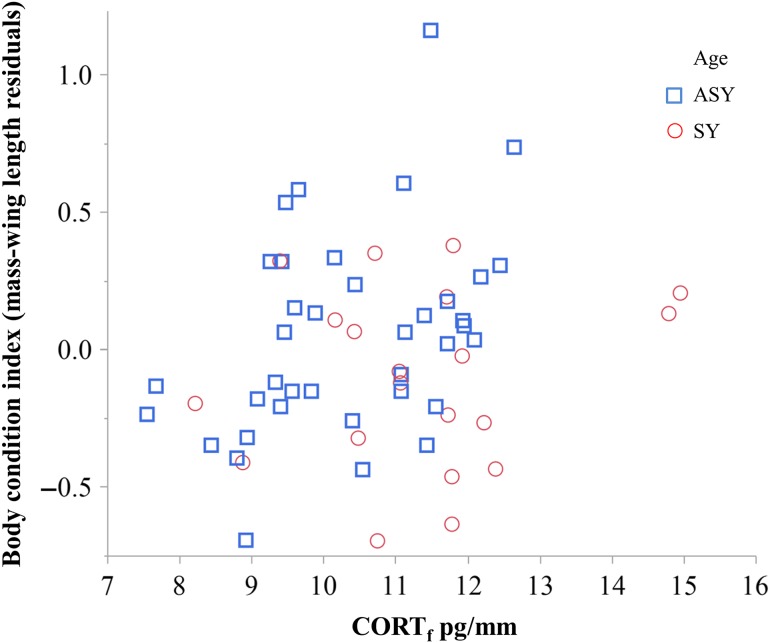
The concentration of corticosterone in male cerulean warbler rump feathers (CORT_f_) grown during non-breeding was positively related to body condition while controlling for age and year (*F* = 5.61, d.f. = 1,54.9, *P* = 0.02) during the subsequent breeding season in the Cumberland Mountains, TN, USA, during 2008–10. ASY, after-second-year; SY, second-year.

Body mass at capture was positively related to rump CORT_f_ (*F* = 4.21, d.f. = 1,53.9, *P* < 0.05), after controlling for the effect of age (ASY males were heavier than SY males; *F* = 14.83, d.f. = 1,52.8, *P* = 0.0003). Likewise, body mass residuals were positively related to rump CORT_f_ (*F* = 5.61, d.f. = 1,54.9, *P* = 0.02; Fig. 3), after controlling for the effect of age (*F* = 3.47, d.f. = 1,54.1, *P* = 0.07). The rump CORT_f_ × age interaction term was not significant in either model (both *P* > 0.41) and was removed from both final models.

### Relationship between non-breeding feather corticosterone and fitness parameters

We found no relationship between rump CORT_f_ and any of the reproductive or survival metrics (while controlling for age and year; Table 1). All CORT_f_ × age interaction terms were non-significant (all *P* > 0.24), and territory basal area had no effect on inference for any variable (all *P* > 0.56), so both terms were removed from all final models.

**Table 1:** Results from general linear models evaluating the relationship between predictor variable of rump corticosterone and various body condition and fitness-related response variables in male cerulean warblers (*Setophaga cerulea*) captured in the Cumberland Mountains, TN, USA, 2008–10

Response variable	*n*	β estimate	*F*-value	*P*-value
Body condition index	59	0.07	5.61	0.02*
First egg date	43	−0.03	1.65	0.21
No. of fledglings produced	49	−0.004	0.01	0.94
Pairing status (Y/N)	58	−0.18	0.74	0.39
Nest success (Y/N)	54	0.02	0.01	0.91
Provisioning rate (visits/h per nestling)	23	−0.10	0.42	0.52
Apparent annual survival (Y/N)	60	0.18	0.65	0.42

All models controlled for age and year (random). Body condition index also controlled for date of capture (random; see Materials and methods). An * indicates statistical significance.

## Discussion

Non-lethal carry-over effects from previous seasons and geographical locations have recently been suggested as important contributors to individual fitness differences and population declines in species that undergo seasonal migrations (Norris *et al*., 2004; Norris and Marra, 2007; Harrison *et al*., 2011). Corticosterone has been implicated as a physiological mediator of these seasonal interactions (Fairhurst *et al*., 2015b; Harms *et al*., 2015), and our study explored this idea using feathers grown during two different life-history stages (post-breeding moult and wintering moult) in younger (SY) and older (ASY) male cerulean warblers. In general agreement with our first two predictions, we found that SY birds had overall higher CORT_f_ concentrations than ASY individuals at both moult stages (and there was no individual consistency in CORT_f_ from post-breeding to wintering), and CORT_f_ was related (positively) to body condition during the subsequent breeding season, and this relationship appeared to be stronger in ASY birds. Despite these relationships, individual CORT_f_ concentrations were not related to any measure of future fitness (contrary to our third prediction). These results suggest that these birds may have benefitted from elevated CORT levels (e.g. via hyperphagia), but this depends on life-history stage, and these short-term benefits may not necessarily affect overall reproduction and survival. We discuss the implications of each of these results below.

### Age and year

Our results provide important age-specific data on CORT_f_ in a Neotropical migrant songbird. Young birds are naïve to many of the challenges that they face during their first year of life. Thus, birds undergoing their first migration and wintering in unfamiliar locations may be relegated to lower-quality wintering habitats and may not be able to cope with adverse environmental conditions as well as experienced adult birds. Accordingly, younger cerulean warblers had higher concentrations of CORT_f_ than older birds during both moulting periods and in most years. These results are consistent with the constraints associated with the first year of life for migratory songbirds (e.g. McKim-Louder *et al*., 2013), particularly during migration, when the greatest mortality rates are believed to occur (Sillett and Holmes, 2002; Klaassen *et al*., 2014).

The age-based pattern of CORT_f_ levels could also be explained by life-history theory, whereby older birds (that have fewer opportunities for future reproduction) may suppress their stress response to ensure no hormonal interference with, and conserve energy for, their limited remaining opportunities for reproduction (Wingfield and Sapolsky, 2003; Lendvai *et al*., 2015). However, even though young birds displayed higher concentrations of CORT_f_ overall, this relationship varied somewhat by year (particularly for feathers grown on the wintering grounds). This demonstrates flexibility in CORT physiology and suggests that local conditions on wintering grounds determine CORT responses as much as, or even more so, than life-history stage. Individual-based differences in CORT physiology would also fail to explain the relationship between age and CORT_f_, because CORT concentrations in feathers grown at different times of the year were not correlated within birds. We would expect CORT concentrations in feathers from the same individual grown in different environmental conditions to be correlated if they were largely determined by endogenous (rather than environmental) factors. Sampling the same birds in multiple seasons and over multiple years would be necessary to show a true lack of consistency, but our results suggest individual flexibility from year to year and between life-history stages within a year (similar to Ouyang *et al*., 2011). In addition, because cerulean warblers are migratory and the CORT physiology that we measured preceded breeding by several months, a life-history strategy (related to age and reproduction) explaining the age-based pattern of CORT seems unlikely.

Annual fluctuations in weather (and associated food availability) could explain the annual variation in CORT_f_ that we detected. Of particular importance to cerulean warblers on the wintering grounds may be the El Niño–Southern Oscillation phenomenon, which can have profound effects on weather across the Andes (Garreaud 2009). During the cold ocean phases (La Niña), air temperatures are depressed and precipitation is increased in the northern Andes. If in place for long time periods, these conditions may lead to decreased insect food availability (relative to short-term La Niña or El Niño events; Van Bael *et al*., 2004). We recorded relatively high levels of CORT_f_ from rump feathers of birds captured in 2009, which followed 2 years of La Niña conditions (National Weather Service, 2015). Although suggestive of an effect of food availability on CORT physiology, more research on this topic is warranted.

### Body condition

Chronically elevated levels of CORT are typically associated with adverse environmental influences and can have detrimental effects on individuals (Romero, 2004; Bonier *et al*., 2009; Blas, 2015). For example, in another closely related Neotropical–Nearctic migrant, the American redstart, elevated CORT is associated with poor-quality winter habitat in Jamaica (Marra and Holberton, 1998) that carries over to the breeding season and is linked to poor body condition (Marra *et al*., 1998). However, in cerulean warblers, the reverse was true; relatively high CORT_f_ from winter was correlated with improved body condition on the breeding grounds. This pattern appears to be driven mostly by ASY males (despite a lack of significant interaction with age), which had lower CORT overall so older birds may be better able, physiologically, to capitalize on the benefits of elevated CORT (e.g. increased foraging; see Fairhurst *et al*., 2014). Unlike territorial American redstarts in Jamaica, cerulean warblers appear to be, at most, semi-territorial during the winter, and are often associated with mixed feeding flocks (Jones *et al*., 2002; Muñoz and Colorado, 2012). Thus, less competitive exclusion may occur in cerulean warblers, and habitat-driven CORT physiology may not be as crucial as it could be for more territorial species that may be relegated to a relatively small space on the wintering grounds (such as American redstarts in Jamaica).

The fine-scale timing of moult could help explain the characterization of elevated CORT as a beneficial physiological response (as may be inferred by the positive relationship between CORT and body condition, particularly for older birds) or as an indicator of poor foraging conditions/abilities (as may be inferred from the SY birds having higher CORT). Although broad patterns of moult in this species are now fairly well understood, the exact timing and variability within and among age classes is unknown. Thus, age-specific differences in the timing of the moult could further confound our ability to discern the underlying relationships (see Romero and Fairhurst, 2016 for a discussion). If pre-alternate moult occurs closer to the end of the winter season in older birds (e.g. March) when individuals are preparing physiologically for a costly migration, higher CORT concentrations under any food availability regime may reflect the pre-migratory benefits of elevated CORT (e.g., hyperphagia; Lõhmus *et al*., 2006). However, if moult occurs earlier in winter, regardless of age, when food limitation may be less likely, elevated CORT levels might reflect occupancy of low-quality habitats or poor food availability. Moreover, if the early moult scenario is the case, the period reflected by CORT_f_ measurements would be even further removed from the future breeding period during which we looked for evidence of carry-over effects, making CORT–fitness relationships even less likely (see next subsection).

### Fitness

Despite its relationship with breeding body condition, we found no evidence that non-breeding CORT_f_ was related to future fitness. Although physiological responses to environmental conditions on the wintering grounds may mediate how cerulean warblers transition from non-breeding to breeding, events on the breeding grounds appear to be more influential to reproduction (e.g. Boves *et al*., 2013). This interpretation is congruent with recent evidence from 19 migratory bird species in the UK, where climatic variation on the breeding grounds had a much greater influence on reproductive performance than did winter climate (Ockendon *et al*., 2013), and with results from a study that found a lack of influence of non-breeding conditions on future fitness of winchats (*Saxicola rubetra*) wintering in Africa and breeding in Europe (Blackburn and Cresswell, 2016). Previous evaluations of CORT_f_ from non-breeding birds in the context of carry-over effects have also failed to find relationships with reproductive output within a single year (e.g. in great skuas, *Stercorarius skua*; Bourgeon *et al*., 2014), but others have found relationships across several years (e.g. in common eiders, *S. mollissima*; Harms *et al*., 2015) or with specific reproductive traits related to condition and feeding (Kouwenberg *et al*., 2013). Had we evaluated fitness carry-over effects in females (which are very difficult to capture), we might have been more likely to find a relationship between CORT and reproduction, because costs are typically greater for females. However, the relationship between winter habitat quality and reproduction is stronger in males for American redstarts (Rushing *et al*., 2016), possibly because reproductive output is more variable in males.

Combining our results with those of others, the role of CORT in carry-over effects appears to be species and context specific. In cases where strong selection occurs at each stage of the life/annual cycle, carry-over effects may be weak or absent (Senner *et al*., 2014), so the relative influence of CORT from non-breeding birds on future reproductive performance may be minimal. Of course, we were able to consider carry-over effects only on those birds that survived the rigours of spring migration after their pre-alternate moult. Thus, we cannot rule out the possibility that environmental conditions from previous seasons still could have induced carry-over effects. For example, prior environmental conditions may have influenced the likelihood of birds surviving the spring migration north, because migration is where most mortality of migratory songbirds is thought to occur (Sillett and Holmes, 2002) and stopover locations may be more poorly protected than breeding habitat (Mehlman *et al*., 2005; but see Runge *et al*., 2015). However, this hypothesis is untestable in this species because direct long-distance tracking of cerulean warblers (or other small migrants) during migration is not feasible with current technology. If migration is the life-history stage of greatest mortality in cerulean warblers, carry-over effects from winter may be limited to influencing survival to the breeding grounds and condition upon arrival. Individuals that do survive the winter and spring migration may arrive at their breeding grounds in a variety of physiological states, but local conditions may rapidly counteract residual effects from the non-breeding season. Thus, as long as a bird can complete the journey from South America to their North American breeding grounds, carry-over effects from wintering grounds, although potentially mediated by CORT, may be relatively short lived and have little bearing on future fitness. Studies of other species that are able to be easily and rapidly captured early in the breeding season and that allow for individual arrival dates to be estimated with certainty (e.g. prothonotary warblers, *Protonotaria citrea*), when potential carry-over effects may still manifest, will be helpful for testing this hypothesis.

### Conservation implications

Although CORT_f_ from the winter is useful for understanding the state in which cerulean warblers transition into breeding, and can be related to individual condition during breeding, we found no support for the hypothesis that CORT physiology from the winter carries over to affect future reproduction or survival to the extent that has been documented for some species (e.g. American redstarts). Instead, individual fitness and, scaling up, population trends of cerulean warblers may be driven to a greater extent by conditions on the breeding grounds (including forest management and landscape-level fragmentation; Thompson *et al*., 2012; Boves *et al*., 2013a, b). Fitness-related seasonal interactions may still exist, particularly from the winter to migration, but these interactions will be difficult to document in the near future. Nevertheless, our study revealed age-specific variation in CORT_f_ during winter; therefore, understanding age-specific ecology of cerulean warblers on their wintering grounds will shed light on what specific factors drive this variation and possible carry-over effects in this species. We also suggest that future studies estimate arrival date on breeding grounds and condition upon immediate arrival because, as potentially important determinants of breeding success, these factors might be relevant to CORT-mediated carry-over effects.
